# Kinetic data for modeling the dynamics of the enzymes involved in animal fatty acid synthesis

**DOI:** 10.1042/BSR20222496

**Published:** 2023-07-19

**Authors:** Chilperic Armel Foko Kuate, Oliver Ebenhöh, Barbara M. Bakker, Adélaïde Raguin

**Affiliations:** 1 Institute for Quantitative and Theoretical Biology, Biology department, Heinrich Heine University, Düsseldorf, Germany; 2 Institute for Computational Cell Biology, Computer Science department, Heinrich Heine University, Düsseldorf, Germany; 3 Cluster of Excellence on Plant Sciences (CEPLAS), Heinrich Heine University, Düsseldorf, Germany; 4 Institute for Systems Medicine of Metabolism and Signaling, Pediatrics department, University of Groningen, University Medical Center Groningen, Groningen, The Netherlands

**Keywords:** Enzyme kinetics, Fatty acid synthesis, Mathematical modeling, Metabolism

## Abstract

The synthesis and modification of fatty acids (FAs) from carbohydrates are paramount for the production of lipids. Simultaneously, lipids are pivotal energy storage in human health. They are associated with various metabolic diseases and their production pathways are for instance candidate therapeutic targets for cancer treatments. The fatty acid *de novo* synthesis (FADNS) occurs in the cytoplasm, while the microsomal modification of fatty acids (MMFA) happens at the surface of the endoplasmic reticulum (ER). The kinetics and regulation of these complex processes involve several enzymes. In mammals, the main ones are the acetyl-CoA carboxylase (ACC), the fatty acid synthase (FAS), the very-long-chain fatty acid elongases (ELOVL 1–7), and the desaturases (delta family). Their mechanisms and expression in different organs have been studied for more than 50 years. However, modeling them in the context of complex metabolic pathways is still a challenge. Distinct modeling approaches can be implemented. Here, we focus on dynamic modeling using ordinary differential equations (ODEs) based on kinetic rate laws. This requires a combination of knowledge on the enzymatic mechanisms and their kinetics, as well as the interactions between the metabolites, and between enzymes and metabolites. In the present review, after recalling the modeling framework, we support the development of such a mathematical approach by reviewing the available kinetic information of the enzymes involved.

## Introduction

Fatty acids (FAs) are of utmost biological importance for living systems. Their synthesis pathway varies among plants, bacteria, and animals [1,2]. They are building blocks for lipids and the primary form of energy storage. Besides their energy-related role, FAs are the main constituents of cellular and organellar membranes, precursors for several signaling pathways, and play an anti-inflammatory role [3,4]. In animals, recent investigations suggest their importance as therapeutic candidates for various disorders. They are involved in cancer [5,6], nonalcoholic fatty liver disease [7], type II diabetes [8], obesity, and inherited metabolic conditions such as fatty acid oxidation disorders [9,10]. Considering the central role of FAs for human medical and nutritional applications, we focus on animals with particular attention on mammals. Mathematical and computational modeling has become increasingly popular and successful in the investigation of pathway dynamics, as it allows to decipher complex systems, and interpret counter-intuitive observations [11]. It includes both static methods (e.g., genome-scale flux balance analysis, atom mapping, and network topology analysis) and dynamic ones (e.g., stochastic or deterministic modeling, and stable isotope tracers) [12–17].

The present review is addressed to mathematical system biologists who aim to model the dynamics of animal fatty acid synthesis using ordinary differential equation (ODE) models based on kinetic rate laws, a typical deterministic approach. The kinetic rate laws and associated parameters for the FA β-oxidation are largely available in the literature [18–25] as demonstrated by the model of van Eunen and coworkers [24]. Yet, analogous information in the case of fatty acid synthesis is scarce. We, therefore, choose to focus on the latter and review the kinetic features of the key enzymes involved in the main pathways of animal fatty acid synthesis (mainly mammals). This includes fatty acid *de novo* synthesis (FADNS), and microsomal modifications (elongation and desaturation) for both endogenous and exogenous FAs. Noticeably, we do not report on the mitochondrial fatty acid synthesis, and we neglect enzymes and transporters that have only a secondary impact on the fatty acid synthesis, e.g., the malonyl-CoA decarboxylase, the acyl-CoA, and the fatty acid-binding proteins. The first section smoothly introduces a nonspecialist reader to the modeling framework, and motivates the latter in comparison with other methods. Then, we individually present the enzymes involved and summarize their kinetic information necessary for modeling. In the Discussion and Conclusions section, we highlight potential new research directions that still require further investigations. In the Supplementary Material section, we provide tables with kinetic parameters and the corresponding rate laws.

### Nomenclature

Two distinct nomenclatures of FAs are spread in the field, i.e., delta and omega. Throughout the paper, we use the latter one. Then, *X:Yn-Z* stands for a FA with *X* carbon atoms and *Y* double bonds (*X:0* describes a saturated FA), and *Z* is the position of the first double bond counted from the methyl end. Because of the way they are introduced, double bonds appear with a spacing of three carbon atoms. Most naturally occurring FAs have an even number of carbon atoms.

## Enzyme kinetic modeling using rate laws

### The framework

Metabolism is the set of chemical reactions that allows organisms to perform their biological functions. As it involves thousands of enzymes, it is divided into pathways, for instance, defined as the ensemble of reactions leading to the production or the consumption of particular compounds. Pathways can be studied from a static or a dynamic point of view, which is chosen depending on the size of the pathway, the questions to be addressed, the pre-existing information, and the assumptions. Static modeling, such as stoichiometric models and constraint-based models, allows studying the distinct metabolic routes and the associated fluxes at the steady states [12,26]. They are particularly relevant for large-scale models (e.g., genome-scale) that involve up to thousands of reactions and metabolites. However, if one is interested in tracking metabolites change over time, dynamical models are required. Furthermore, access to the concentration of the system’s metabolites over time allows to discriminate optimal fluxes predicted by static models that may pass through biologically unrealistic transient states. Among dynamic models, one could differentiate between deterministic and stochastic ones. The latter specifically allow to study systems made of a small number of entities, their fluctuations, and to represent *in silico* large and complex substrates for which kinetic rate laws cannot be derived (e.g., glycogen). In the present review, we focus on the FA biosynthesis pathway and provide biochemical information to construct models simulating its dynamics based on kinetic rate laws. Thereby, one can study metabolic pathways both qualitatively and quantitatively. Kinetic rate laws define the velocity at which a chemical reaction occurs while highlighting the underlying mechanism, the contribution of each element participating in the reaction, and their interactions [27,28].

The rate of change over time of individual chemical entities (noted *M_i_*) is expressed as the sum and difference of rate law terms (noted $v_{j}$), weighted by their stoichiometric coefficients (noted *α_ij_*), following (eqn 1.1):

(1.1) $$\frac{dM_{i}}{dt}=\sum_{j}\alpha_{ij}v_{j}$$

A good overview of some simple and commonly used kinetic rate laws has been published by Saa and Nielsen [29], as well as Kim et al. [12]. The closer a reaction is to the equilibrium, the least details are needed to model it. Kinetic rate laws require parameters that can be estimated from experimental data using methods such as Lineweaver-Burk plots [30–32], Hanes-Woolf plots [33], and nonlinear regressions [34]. Still, when missing kinetic parameters, the typical approach consists in either using those from a related organism, or implementing simplistic rate laws such as mass-action kinetics or its generalization. Although these types of rate laws are easy to analyze, they have the disadvantage of not capturing substrate saturation [35]. To circumvent this limitation, more complex kinetics can be used, such as Michaelis–Menten [36], multisubstrate (sequential or ping-pong) [27], ‘convenience’ [35] or ‘universal’ [37] kinetics. They account for the order of addition of the substrates, the order of formation of the products, the different types of inhibition, the activations, the competitions, etc. Aside from these strengths, the potentially large number of parameters is usually an obstacle for further analysis. Besides, the King and Altman method is a generic approach to derive kinetic rate laws, step-by-step, from the underlying specific enzymatic mechanism [38–40]. It allows determining the kinetic rate laws of complex mechanisms involving one or more reactants, products, inhibitors, etc. The steps of the method have been fully described by Cleland et al. [27] and Ulusu et al. [41]. Simpler kinetics such as Michaelis–Menten can also be retrieved by this means. In addition, the Haldane relationship can be used to relate the kinetic parameters and the thermodynamics of the reaction via the equilibrium constant.

Generally, the mathematical representation of the dynamics of the pathway leads to large systems of nonlinear differential equations that are difficult or impossible to solve and interpret analytically. Therefore, dedicated software tools have been developed to solve them numerically. Among others, we can mention Modelbase [42], COPASI [43], PySCeS [44], and Berkeley Madonna [45].

### Aims of the method

Kinetic models can help testing hypotheses, designing and assisting experiments, discriminating between possible regulatory mechanisms, identifying drug targets, and make sense of genomic, proteomic, and metabolic data [29,46–48]. They allow predicting how the system responds to perturbations, or identify the existence of rescuing pathway routes, for example, toward understanding disease phenotypes. They also can be used to predict the time course of metabolite concentrations and fluxes, for instance, toward optimizing the latter. For illustration, we report here a typical example where theory-derived hypotheses have been verified experimentally and led to major progress in understanding the associated metabolic pathway. It is known that hypoglycemia observed in medium chain acyl-CoA dehydrogenase deficiencies is the result of the inability of *β*-oxidation to deal with substrate influx, because one of the first enzymes involved in this cyclic pathway is defective. Remarkably, using a detailed kinetic model, based on a bottom-up approach and a set of mice experiments, Martines et al. [10] have been able to predict that intermediate metabolites accumulate within the pathway, causing the reverse reactions to be more thermodynamically favorable than the forward ones. Using metabolic control analysis, they also showed that medium-chain ketoacyl-CoA thiolase (MCKAT) can restore the pathway fluxes, revealing its potential for being a drug target. As an incentive, we additionally present two specific cases of experimental observations that would typically benefit from a complementary kinetic modeling investigation. First, experimental results by Santos and Schulze [49], and de Cedrón and de Molina [50], showed that targeting acetyl-CoA carboxylase (ACC) and fatty acid synthase (FAS), or adopting appropriate diet habits, holds the potential to reduce tumor growth, since long-chain-saturated fatty acids (LCSFAs) are considered as a fuel for tumor proliferation [51,52]. Beyond this qualitative observation, the quantitative details of these therapeutic approaches are not specified, nor their consequences on the whole lipid metabolism. A kinetic model could help to quantitatively characterize the respective contribution of each enzyme to the overall LCSFAs *de novo* production, thereby unveiling their potential as drug target. One should note that, although Menendez and Lupu [52] showed that FADNS is more pronounced in tumor cells, tumor proliferation is a complex process that involves several factors (see the review by Fhu and Ali [53]). Second, in FA metabolic disorders, such as obesity and type 2 diabetes, Lelliott and Vidal-Puig [54] observed lipotoxicity caused by an imbalance between *de novo* lipogenesis and FA oxidation. To counteract this effect, the authors suggested to both decrease the fat deposition in the white adipose tissues, and to increase the FA oxidation capacity of oxidative tissues. To test this hypothesis, one could develop a lipid metabolism model that includes both lipogenic and oxidative tissues, as well as, all biochemical reactions relating to FA metabolism and their hormonal regulation, at the whole body level. Furthermore, the model should allow to quantify the FA fluxes and concentrations in each tissue in a time-dependent manner. To fulfill these requirements, and ensure that the theoretical parameter values resulting from the model do not correspond to unrealistic biology, e.g., thermodynamically unfeasible, a kinetic model appears most appropriate.

## FADNS

The FADNS, also known as the endogenous synthesis of FAs, produces LCSFAs from acetyl-CoA in presence of ATP, bicarbonate (${\text{HCO}}_{3}^{-}$), and NADPH. The process can be divided into two parts: (i) the synthesis of malonyl-CoA from acetyl-CoA by the biotin enzyme ACC, and (ii) the step-wise elongation of the acyl-CoA chain by the multicomplex enzyme FAS. The main resulting product is palmitic acid (16:0), together with some myristic (14:0) and stearic (18:0) acids, and very low amounts of lauric (12:0) and arachidic (20:0) acids [55–57]. Below, we provide a brief introduction to the biochemistry of each enzyme. For further details, the reader is referred to [58,59] for ACC and [1,2,60] for FAS. In addition, for the reader to construct their models, we tabulated the kinetic rate laws associated to the enzymes (see Table 1). To keep our tables short, we focus on the simplest kinetic laws. For the more complex ones, we refer the reader to the original articles discussed in the text. We also gathered the associated kinetic parameters (see Tables 2 and 3). Several blanks appear in both tables, highlighting some of the gaps in the data availability.

**Table 1:** Kinetic rate laws corresponding to the parameters reported in Tables 2, 3, and 6–8

Enzyme	Rate law	Formula	Parameters	Reference
ACC	Competitive inhibition and activation	$V_{max}\times S/(K_{m}(1+I/K_{i})+S(1+K_{a}/A))$	Table 2	[68]
ACC ELOVL Δ-9, Δ-5, Δ-6	Michaelis–Menten kinetics	$k_{cat}\times E_{0}\times S/(K_{m}+S)=V_{max}\times S/(K_{m}+S)$	Tables 2, 3, and 6–8	[66,89,92,108–110,122–124,126–128,134]
FAS		$k_{cat}\times E_{0}/(1+K_{M_{M\;al}}/M\;al(1+Acet/K_{I_{Acet}})+K_{M_{A\;cet}}/Acet\;(1+M\;al/K_{I_{M\;al}})+K_{M_{NADPH}}/NADPH)$	Table 3	[34]

**Table 2:** Kinetic parameters of ACC isoenzymes

ACC	
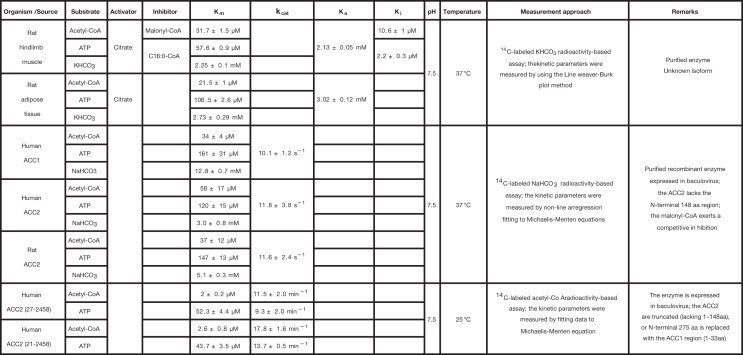	**Reference**
	[68]
	[66]
	[134]

Cells are empty when the parameter values were either not measured or not considered in the rate laws. The meaning of each symbol is defined in the List of Symbols.

**Table 3:** Kinetic parameters of FAS

FAS & active sites	
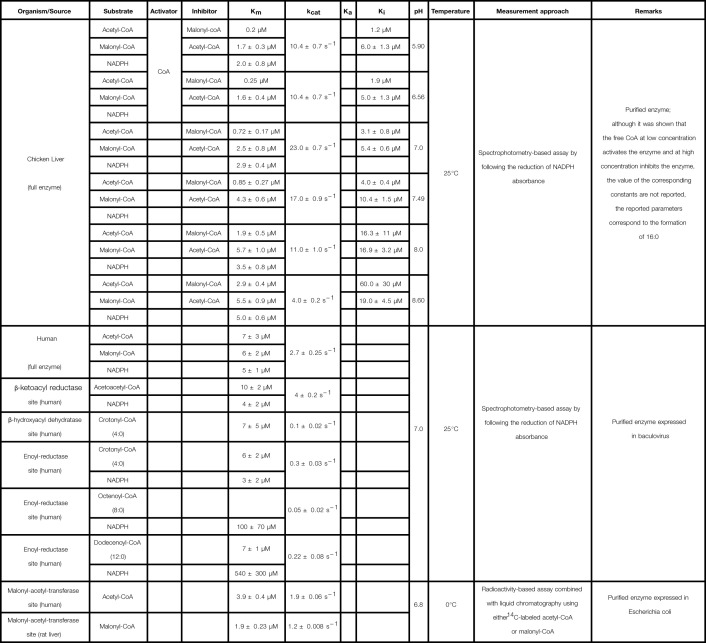	**Reference**
	[34]
	[92]
	[89]

Cells are empty when the parameter values were either not measured or not considered in the rate laws. The meaning of each symbol is defined in the List of Symbols.

Although some of the kinetic features discussed here are not from mammals, they can still be used as a starting point for modeling. They typically share a similar enzymatic mechanism, as well as high amino acid sequence identity. For instance, chicken and rat FAS sequences are 63% and 79% identical to that of human, respectively [61]. Besides, among mammals, it is observed that murine FAS sequence is 81%, 78%, and 94% identical to that of human, pig, and rat, respectively [33].

### ACC

**Figure 1:**
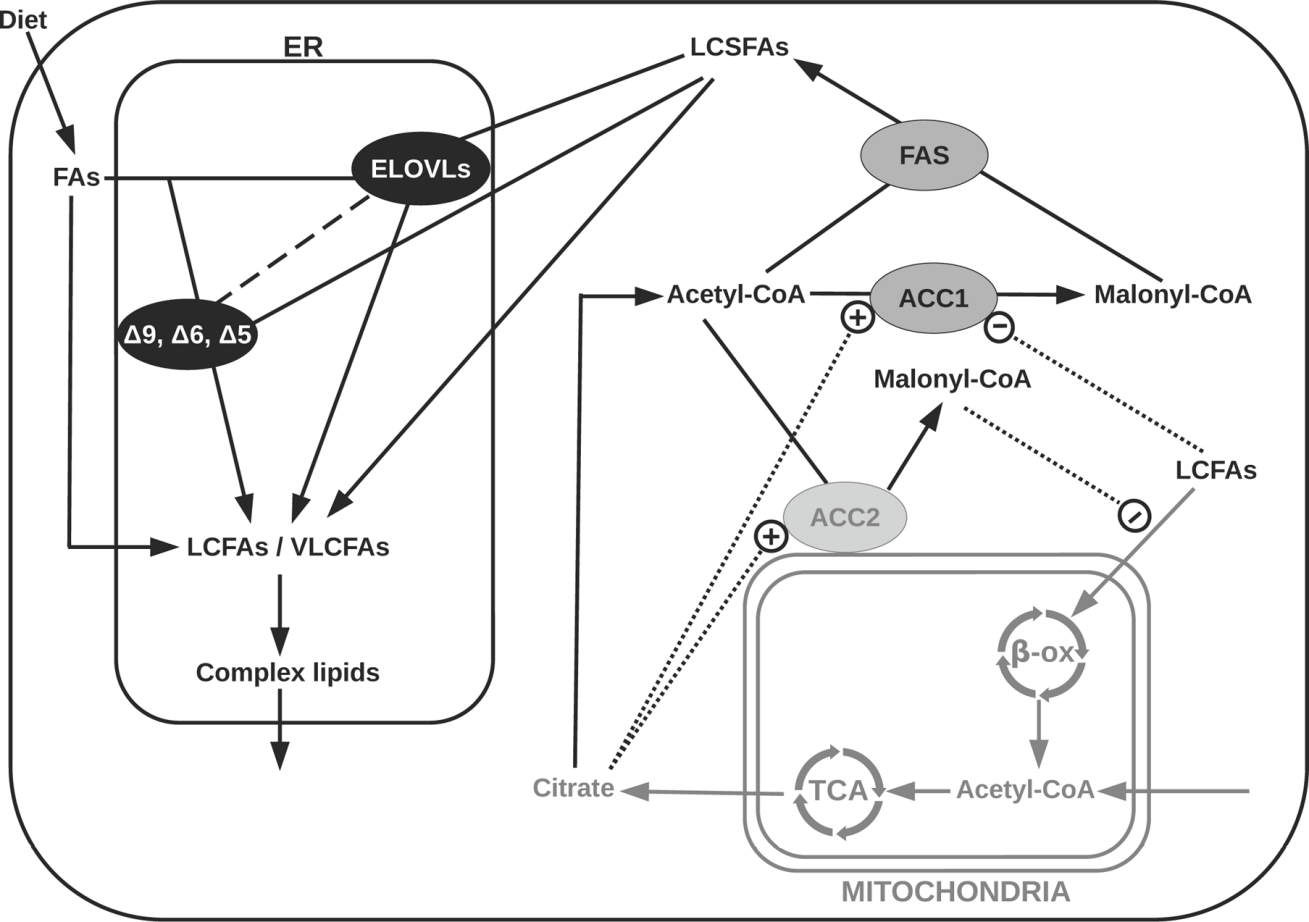
Schematic representation of the biochemistry of fatty acid biosynthesis The process is organized in two main parts. Enzymes involved in the FADNS (FAS and ACC1) are color-coded with a gray background. They are responsible for the production of long chain saturated fatty acids (LCSFAs). This process takes place in the cytoplasm. Enzymes involved in the microsomal modifications of fatty acids (ELOVLs, Δ-desaturases) are color-coded with a black background. They are responsible for elongating and desaturating long-chain fatty acids (LCFAs) and very long-chain fatty acids (VLCFAs). This process takes place in the endoplasmic reticulum (ER) where these enzymes are membrane-bound. In the ER, LCFAs and VLCFAs, represented on the figure, include LCSFAs, monounsaturated FAs (MUFAs), and polyunsaturated FAs (PUFAs). The β-oxidation that takes place in the mitochondria is not part of the fatty acid synthesis. Still, it is represented because it influences the overall synthesis process. For the sake of simplicity, several enzymes and transporters that are not directly involved in the synthesis are not represented, e.g., malonyl-coA decarboxylase, fatty acid-binding proteins, and acyl-CoA-binding proteins.

ACC is a key enzyme for lipid homeostasis [62,63]. To date, two isoforms of ACC are known (i.e., ACC1 and ACC2). They are encoded by two distinct genes that share 80% of similarity when comparing their amino acid sequences. One of the major differences between the two isoforms resides in their N-terminal amino acid sequences [64]. For ACC2, the latter starts with hydrophobic residues that are responsible for its membrane-bound properties and its location at the surface of the mitochondrial outer membrane [64,65]. Opposite, ACC1 is a soluble cytosolic enzyme (see Figure 1). Despite these differences, ACC1 and ACC2 share a similar mechanism, and noticeably, they are reported with comparable kinetic parameter values (see Table 2) [66–68]. More precisely, we can remark that in the study by Cheng et al. [66], for human ACC1 and ACC2, the *k*_cat_ values measured are almost indistinguishable (when considering the standard errors) and the *K*_m_ values show very similar tendencies for the three substrates considered. In the same study, the authors observed comparable values for ACC2 in rat. In addition to show distinct location in the cell, ACC1 and ACC2 have different tissue-specific expression. The ACC1 isoform, which is found in all tissues, is particularly highly expressed in lipogenic tissues such as the liver, adipose tissues, and mammary glands. The second isoform, ACC2, is mostly present in oxidative tissues such as the heart and the skeletal muscles. It is also expressed, to a lesser extent, in lipogenic tissues [69–71]. In the respective tissues, the malonyl-CoA produced by ACC1 is utilized for elongation in FADNS, while the one from ACC2 also inhibits the carnitine palmitoyl transferase 1 and thereby the *β*-oxidation [65,72].

Both ACC isoforms synthesize malonyl-CoA from acetyl-CoA in three steps. First, the ACC is carboxylated, using ${\text{HCO}}_{3}^{-}$ as carbon donor in presence of ATP. Second, the carboxyl group is transferred between the catalytic sites of the enzyme. Third, the carboxyl group reacts with acetyl-CoA to form malonyl-CoA [66]. Citrate, exported from mitochondria, is converted in cytosolic acetyl-CoA, which is further converted into malonyl-CoA by ACC1 and ACC2 enzymes. The reaction catalyzed by ACC2 is itself activated by citrate (see Figure 1) [60]. By activating ACC2, citrate also indirectly inhibits the *β*-oxidation, and thereby positively regulates the FADNS pathway [73,74]. Besides, the ACC enzymes are allosterically repressed by malonyl-CoA, long-chain fatty acyl-CoA, and free CoA [66,75,76]. Both ACC isoforms are also subject to diet and hormonal regulations [60,77–79]. In addition, magnesium, in the form of Mg^2+^, facilitates the transfer of the carboxyl group of the ATP to the biotin coenzyme, and stabilizes the transition state during the first step of the malonyl-CoA synthesis reaction (carboxylation reaction) [80]. Thus, Mg^2+^ is a cofactor for the reaction and it influences its kinetics.

Our current understanding of the ACC mechanism is based on several studies that have been reviewed by Numa and Tanabe [74]. Using isotope labeling, the three steps of the mechanism could be characterized. Also, Hashimoto et al. [81] suggested an ordered Bi Bi Uni Uni Ping Pong mechanism with an activation by citrate. The order of attachment of the substrates to the enzyme is, ATP, ${\text{HCO}}_{3}^{-}$, and acetyl-CoA for the forward reaction, and malonyl-CoA, Pi, and ADP for the reverse reaction [81]. The reaction is subject to product regulation, notably by malonyl-CoA and ADP. The mathematical expression of the rate law, including numerical values of the kinetic parameters, has been reported by Hashimoto et al. [81]. With 16 parameters, it is quite complex, although it does not include inhibition by long-chain acyl-CoAs. The reaction mechanism was later questioned by Beaty and Lane [82], and Kaushik et al. [67], who instead proposed the random Ter Ter mechanism. Both articles provide detailed kinetic parameter values, useful for the construction of models. Liquid chromatography/mass spectrometry/mass spectrometry (LC/MS/MS) data of malonyl-CoA formation were fitted to the proposed rate law, allowing characterizing the human recombinant ACC2 [67]. The resulting kinetic parameters resemble those reported for rat skeletal muscle [68] and human recombinant enzymes [66]. Besides, Ogiwara et al. [75] and Tanabe et al. [83] focused on the inhibition constants of natural inhibitors (both substrate and product inhibitions) in rat liver. For example, Tanabe et al. [83] reported the inhibition constants of LCFAs and analogs. Inhibition constants for malonyl-CoA and palmitoyl-CoA were also determined [68].

### FAS

FAS is the cytosolic homodimeric multifunctional enzyme responsible for the channeled elongation reactions in the FADNS [84–86] (see Figure 1). This enzyme comprises seven catalytic sites, including the ACP domain that is responsible for channeling. The reaction is initiated from acetyl-CoA with malonyl-CoA as carbon donor for elongation, and NADPH as reducing equivalent. The elongation cycle takes place in four steps: condensation, reduction, dehydration, and reduction [2]. The thioesterase site releases the final products of the FADNS, mainly 16:0. This suggests a high selectivity of the thioesterase domain for the 16:0-intermediate, which was confirmed by Chakravarty et al. [87] in *in vitro* experiments.

Two main strategies are reported in the literature for the derivation of the FAS kinetic rate law: (i) considering each elementary reaction till the production of a LCSFA [34,88–90]; or (ii) lumping all the steps up to the production of a LCSFA as a single reaction [91,92]. The common feature of the studies using either approach is that acetyl-CoA and malonyl-CoA compete for the same enzyme-binding site.

Studying the mechanism of elongation by focusing on each of the seven enzymatic sites, Katiyar et al. [88] concluded that all the individual reactions follow a Ping Pong mechanism except the reduction steps [88]. The latter were suggested to instead follow random sequential or ordered sequential mechanisms, with NADPH added first, and the proton added second [88]. From these individual steps, the overall kinetic rate law of FADNS was derived using the King and Altman’s method [38]. The resulting detailed rate law has 11 parameters. This complexity may make it difficult to fit the kinetic parameters and to relate them to their biological meaning. In chicken liver, Cox and Hammes [34] proposed a simpler rate law for the overall reaction, following a three substrates Michaelis–Menten kinetics, with competition of acetyl-CoA and malonyl-CoA. They described the associated mechanism with eight elementary steps. The first two correspond to the attachment of acetyl-CoA to the enzyme. Steps 3 to 7 repeat at each elongation cycle, while step 8 is the release of the final product, chosen as palmitic acid (16:0). In their approach, all complex formation involving CoA or NADPH are reversible, characterized by their dissociation constant. Moreover, the authors highlighted the explicit relation between the mechanistic parameters (kinetic rate constants and dissociation constants), and the *k*_cat_ and $K_{m_{i}}$ (*i* is either acetyl-CoA, malonyl-CoA, or NADPH) of the overall kinetics. *k*_cat_ and $K_{m_{i}}$ were measured at various pH-values, thereby highlighting the impact of this experimental condition on the efficiency of the enzyme ($k_{cat}/K_{m_{i}}$).

Other studies employ a twofold approach, to first lump the reactions and derive the rate law, and second measure detailed kinetic parameters for specific reaction steps. For instance, Carlisle-Moore et al. [92] considered a lumped reaction for the production of 16:0 in human, and measured the *k*_cat_ and $K_{m_{i}}$-values (*i* is either acetyl-CoA, malonyl-CoA, or NADPH). Then, the same parameters were determined for the reduction and dehydration steps, when considered separately. In particular, those of the enoyl-CoA reductase site (last one of the elongation cycle) were assessed as a function of the substrate chain length (4:0, 8:0, and 12:0) (see Table 3). A similar approach was followed in chicken liver using tracer experiments in order to determine *V*_max_ and *K*_m_-values for various substrates [91].

*In vitro* measurements of the FAS kinetics suffer from limitations, e.g., the malonyl-CoA decarboxylation into acetyl-CoA, and the natural abundance of the ^13^C carbons that introduces extra noise in the case of ^13^C MS assays. Their impact was observed in kinetic assays by Ohashi et al. [93] and Topolska et al. [57], using enzymes from guinea pig harderian gland and cow, respectively.

## Microsomal modifications of FAs

Metabolic functions require specific FA profiles [94,95]. Some FAs cannot be synthesized *de novo* (essential FAs) and, therefore, must be obtained from external sources. The FAs produced *de novo* (endogenous) and those from the diet (exogenous) are not always suitable, and must be modified accordingly. This modification takes place in the ER and involves two processes, elongation and desaturation. Those are synergized to ensure FA diversity and specific profiles. Elongation produces VLCFAs. They are essential precursors for various classes of lipids, such as phospholipids, sphingolipids, triglycerides, cholesterol esters, and wax esters, whose synthesis are beyond the scope of the present review [96]. Desaturation tunes FA properties [97]. For instance, the cellular and organellar membrane permeability and fluidity depend on the level of unsaturation of their constitutive FAs [98]. Below, we introduce the biochemistry of each enzyme. For the sake of briefness, we also provide tables that summarize the main biochemical characteristics of elongases (see Table 4) and desaturases (see Table 5). For further details, the reader is referred to [99–101] for elongases and [97,99,102] for desaturases. In addition, like in section 2, we tabulated some of the associated kinetic parameters for the reader to construct the respective kinetic rate laws (see Tables 6–8).

**Table 4:** Summary on biochemistry of elongases

Enzyme	Tissue expression	Substrate type	Substrate chain length	References
ELOVL 1	almost all tissues	SFAs and MUFAs	18–26	[96,135–137]
ELOVL 2	testis^+^, liver^+^, brain^−^, kidney^−^, WAT^−^, lung^−^	essential PUFAs, preference for nonessential FAs in mouse	20–22	[96,136–139]
ELOVL 3	skin sebaceous gland, hair follicles, BAT	SFAs, USFAs	16–22	[96,109,136,137]
ELOVL 4	retina, brain, skin, testis^+^, prostate^+^, ovary, thymus^+^, small intestine^+^	SFAs, ULCFAs	≥ 24	[96,136]
ELOVL 5	almost all tissues	essential PUFAs	18–20	[96]
ELOVL 6	almost all tissues	12:0, 14:0, 16:0, 16:1n7, 18:1n9	12–18	[96,138,140,141]
ELOVL 7	brain^−^, liver^−^, small intestine^−^, testis^−^, leukocytes^−^, placenta^−^, colon^+^, kidney^+^, prostate^+^, pancreas^+^, adrenal glands^+^	16:0, 18:0, 20:0, 18:1n9, 18:3n6 preference for nonessential FAs	16–20	[96,109,142]

The symbols “+” and “−” on top of tissues, respectively, mean highly expressed and poorly expressed in the corresponding tissue. If no sign is indicated, the information could not be retrieved from the literature. All acronyms used here are listed in the Abbreviation subsection.

**Table 5:** Summary on biochemistry of desaturases

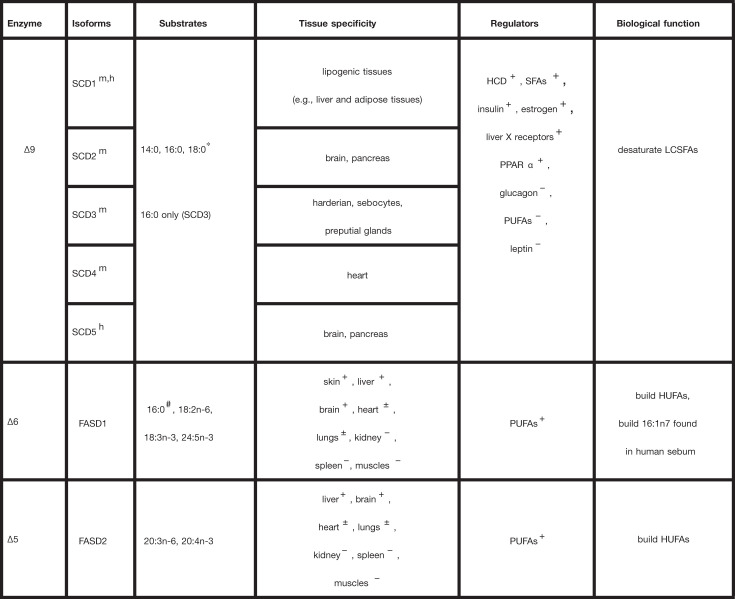	**References**
	[114,118,143–148]
	[114,118,143,148]
	[114,118,143,149]
	[114,118,143,147]
	[114,118,143,148]
	[97,115,116,125]
	[97,115,116,125]

In the isoforms column, *m* and *h* mean present in mice and humans, respectively. In the substrates column, ‘*’ indicates the preferred substrate, while ‘#’ indicates a special case of desaturation. In the tissue specificity column, ‘+’ and ‘−’ indicate that the enzyme is highly or lowly expressed, respectively, while ‘±’ means moderately expressed in the corresponding tissue. In the regulators column, ‘+’ and ‘−’ indicate enzyme activity increase and decrease, respectively. All acronyms used here are listed in the Abbreviation subsection.

**Table 6:** Kinetic parameters of elongases

Elongation cycle/ELOVLs	
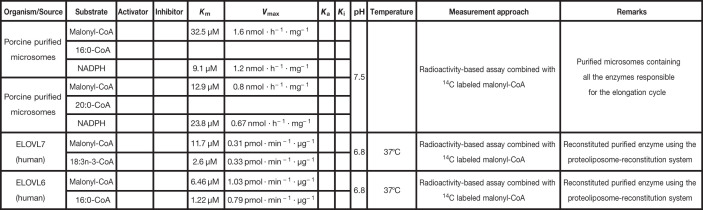	**Reference**
	[108]
	[109]
	[110]

It is important to recall that the purified microsomes are not the purified enzymes. They contain the four enzymes of the elongation cycle, together with other enzymes that could impact their kinetics. The concentration of each elongation enzyme therefore remains unknown. Cells are empty when the parameter values were either not measured or not considered in the rate laws. The meaning of each symbol is defined in the List of Symbols.

**Table 7:** Kinetic parameters of Δ9 desaturase

Δ9 desaturase	
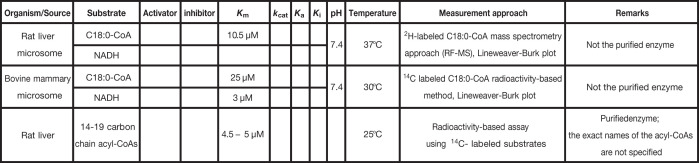	**Reference**
	[122]
	[123]
	[124]

Cells are empty when the parameter values were either not measured or not considered in the rate laws. The meaning of each symbol is defined in the List of Symbols.

**Table 8:** Kinetic parameters of Δ5 and Δ6 desaturases

Δ5 and Δ6 desaturases	
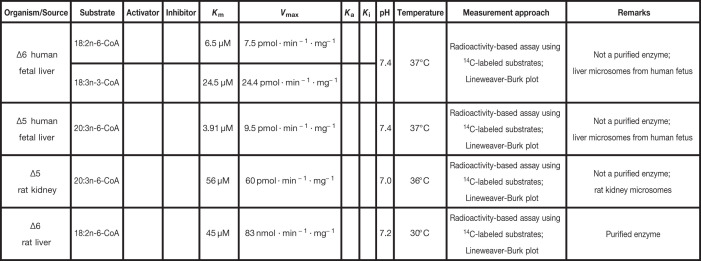	**Reference**
	[127]
	[127]
	[128]
	[126]

Cells are empty when the parameter values were either not measured or not considered in the rate laws. The meaning of each symbol is defined in the List of Symbols.

### Elongases

The microsomal elongation of FAs is the major pathway to produce VLCFAs [103]. Similarly to the *de novo* synthesis, it utilizes malonyl-CoA and NADPH as carbon donor and reducing agent, respectively. The microsomal elongation process consists of four steps: condensation, reduction, dehydration, and reduction. The end product of the elongation is an acyl-CoA with two extra carbons. Unlike the *de novo* synthesis, each step is catalyzed by a distinct enzyme. To date, seven 3-keto-acyl-CoA synthases catalyzing the condensation step have been identified in mammals, the so-called elongation of very long-chain fatty acids (ELOVLs) enzymes [104]. They are membrane bound and located at the surface of the ER (see Figure 1). Besides, unlike the other three enzymes involved in the microsomal elongation process (i.e., 2,3-*trans*-enoyl-CoA reductase, 3-keto-acyl-CoA reductase, and 3-hydro-acyl-CoA dehydratase), the ELOVLs are substrate specific, catalyze the rate-limiting step, and are differently expressed in distinct tissues (see Table 4). Hence, they play a central role in determining the distribution of VLCFAs [96,99,100,104–106], and so we choose to focus on them.

The analysis of the kinetics of the microsomal elongation began with the pioneering work by Nugteren [107] using rat liver microsomes. They ^14^C-labeled malonyl-CoA, long-chain saturated and unsaturated FAs, and intermediates. They studied the overall elongation cycle by deriving the normalized rate of elongation, as a function of the chain length and degree of unsaturation of the substrates. They also reported detailed time-course data for the overall elongation of 14:0 to 16:0. A similar approach was carried out three decades later, using porcine neutrophil microsomes, assuming that the elongation follows a Michaelis–Menten rate law. It was possible to determine *V*_max_ and *K*_m_-values for malonyl-CoA and NADPH for 16:0-CoA and 20:0-CoA, as well as the overall activity for the elongation cycle [108]. Although at that time, ELOVLs were not yet clearly identified, these studies paved the way for investigating their kinetics. Surprisingly, the latter has been little investigated since ELOVLs were identified in the early 2000s. The most popular studies are by Naganuma et al. [109], and Naganuma and Kihara [110] on ELOVL7 and ELOVL6, respectively. In both, the kinetic parameters of the enzyme were determined using HEK 293T cells. For ELOVL7, these were *V*_max_ and *K*_m_-values for malonyl-CoA and 18:3n-3-CoA. For ELOVL6, the corresponding values were determined for malonyl-CoA and 16:0-CoA. Neither ELOVL7 nor ELOVL6 are subject to allosteric inhibition; however, ELOVL6 is repressed by PUFAs [111]. Besides, Naganuma and Kihara [110] showed that NADPH and 3-ketoacyl-CoA reductase enhance the activity of ELOVL7. The underlying mechanism is unknown, but they speculated that the presence of the 3-ketoacyl-CoA reductase may cause a conformational change of the enzyme, thereby increasing its activity. This hypothesis could for instance be tested using fluorescent nanoantennas that allow monitoring small and large protein conformational changes [112].

### Desaturases

The desaturase enzymes are responsible for the introduction of double bonds at specific positions along FA chains. Like the ELOVLs, they are membrane-bound enzymes and are located in the ER (see Figure 1). They are substrate and tissue-specific [99]. Desaturation tailors FA properties (e.g., melting point, rancidity, and flexibility) that ensure their suitability for various biological processes [97]. In mammals, three desaturases have been identified, namely the Δ5 desaturase, the Δ6 desaturase, and the Δ9 desaturase [97,113]. The three desaturations follow the same mechanism. The Δ*X* desaturation consists in introducing a *cis* double bond between the carbons *X* and *X* + 1, counted from the carboxyl end. Via a series of reactions, the Δ*X* desaturase consecutively removes two hydrogen atoms, the first one at the *X*^th^ position, and the second one at the *X* + 1^th^ position [113–116]. These two hydrogens are combined with molecular oxygen and released as water [117]. The electrons required for this reduction are derived from cytochrome *b*_5_ [114–116]. One should note that for unsaturated FAs, a further desaturation does not change the n*Y* family to which the FA belongs, *Y* being the position of the first double bond, counted from the methyl-end. Further biochemistry details of the Δ*X* enzymes are summarized in Table 5, i.e., their isoforms, substrates, tissue specificity, regulators, and biological functions. As for their kinetic features, very little information and data are available. This may be due to particularly challenging experimental tractability. Specifically, here we are dealing with membrane-bound enzymes, whose purification requires several complicated steps. Furthermore, the desaturation reactions involve an intermediate step catalyzed by an extra enzyme (i.e., cytochrome *b*_5_ reductase), making the design of kinetics assays difficult.

#### Δ9 Desaturase

The MUFAs play a crucial role in lipid homeostasis and the physiological functions of lipids [117–119]. To ensure their presence in an adequate proportion, they are endogenously produced from saturated FAs by the Δ9 desaturase, also known as stearoyl-CoA desaturase (SCD). Ntambi et al. [120] and Miyazaki et al. [121] reported that SCD-deficient mice show an increase in insulin sensitivity and are protected against diet-induced adipocity. This suggests that the Δ9 desaturase could be a good therapeutic candidate for obesity and metabolic syndromes. Yet, the literature turns out to be less furnished with respect to the kinetic features of the Δ9 desaturase, as compared with those of the FADNS enzymes. Specifically, the kinetics associated to the detailed mechanism of the enzyme is not discussed. Thus, as a first intention, most studies assume a Michaelis–Menten rate law. Three studies following this approach in mammals can be stressed. They respectively use rat liver microsomes [122], bovine mammary microsomes [123], and purified rat liver enzyme [124], and provide a starting point for kinetic modeling. Since the first two studies do not use purified enzymes, the *k*_cat_ values cannot be determined. As an alternative, one can use the Lineweaver-Burk plot of the kinetic data, together with the mass of the protein, to infer the *V*_max_ values. For example, from the data of Soulard et al. [122], we can estimate the *V*_max_ values to be about 2 μM·min^−1^·mg^−1^ protein and 1.17 μM·min^−1^·mg^−1^ protein, for 18:0 and NADH, respectively. When considering the study by Strittmatter and Enoch [124], it appears that its purpose is not to measure the kinetic parameters, but to present in detail the procedure of purification of the enzyme. Hence, in that case, we can infer neither the *k*_cat_ nor the *V*_max_ values from the reported data. Despite the limited information available in the literature, the reader can take into account the *K*_m_ values tabulated in order to begin developing a kinetic model (see Table 7). We believe that a subsequent effort should be placed into measuring the kinetic parameters of the Δ9 desaturase, notably the *k*_cat_ and *K*_m_ values for the different substrates, and the parameters associated with potential regulatory mechanisms.

#### Δ5 and Δ6 desaturases

The n3 and n6 FA families (also known as ω3 and ω6) are unsaturated essential FA precursors for the synthesis of highly unsaturated FAs (HUFAs). The latter are involved in cell membrane composition, signaling processes, brain and retina development, and cognition and inflammatory responses [97,113]. Animals cannot synthesize *de novo* ω3 and ω6 FAs, but can modify the essential FAs 18:3n3 and 18:2n6 acquired from external sources to fit the adequate fatty acid profiles. The Δ5 and Δ6 desaturases are required for this modification [97,115,116]. Like the ELOVLs and Δ9 desaturase, the Δ5 and Δ6 desaturases are membrane-bound enzymes. Interestingly, the Δ6 desaturase, highly expressed in the skin, has been shown to act on the saturated FA 16:0, resulting in the MUFA 16:1n-10. This special case is highlighted by the symbol # in Table 5. This finding is consistent with the fact that 16:1n-10 is the major FA, found in human sebum [125].

Like for the Δ9 desaturase, the kinetics of animal Δ5 and Δ6 desaturases is understudied. Furthermore, only a few studies use purified enzymes. For instance, Okayasu et al. [126] used the purified Δ6 desaturase from rat liver to measure *K*_m_ and *V*_max_ values for 18:2n-6-CoA. Opposite, Rodriguez et al. [127] focused on the kinetics of both Δ5 and Δ6 desaturases using human fetal microsomes. The *K*_m_ and *V*_max_ values were measured for 20:3n-6-CoA for Δ5, and 18:2n-6-CoA and 18:3n-3-CoA for Δ6. In addition, Irazú et al. [128] measured the *K*_m_ and *V*_max_ values of Δ5 using rat kidney microsomes. For these three studies, a summary of the parameter values, as well as the conditions of measurement, are provided in Table 8. In the table, the kinetic parameters are only reported for essential FAs. One should also note that, although in all these *in vitro* studies the authors report substrate inhibition when the substrates are above a certain threshold, we choose to not tabulate them, since this phenomenon is unlikely to be observed *in vivo*. Finally, nothing is known about the kinetics of desaturation of 16:0 by the Δ6 desaturase.

## Discussion and conclusions

FAs are the precursors of lipid synthesis, and therefore fundamental building blocks in every living cell. It is thus not surprising that many metabolic disorders are associated with defects in FA metabolism [60,129]. Mathematical modeling has become an increasingly popular approach for the investigation of biochemical pathways. Model simulations can guide experiments and support the identification of potential drug targets. Their construction relies on the availability of experimental data concerning the detailed enzymatic mechanisms, the enzyme kinetics, the associated mathematical rate laws, and the values of the corresponding parameters. In the present review, we focused on animal fatty acid synthesis with a particular emphasis on mammals. We aimed to summarize the information necessary for constructing dynamic mathematical models describing this complex enzymatic process using rate laws. We first gave an overview of the framework, and then reviewed the kinetic information of the enzymes involved, including both the FADNS and the microsomal modification pathways. We also provided tables summarizing the kinetic information, as well as the basic biochemistry, of the enzymes involved (Tables 2–8). Throughout, we report facts and figures for each enzyme individually. To calibrate a model encompassing the entire fatty acid synthesis pathway, the reader may also be interested in the *in vivo* data collected by Hems et al. [130] in the liver and the adipose tissues of lean and obese mice.

We find that, despite enormous amount of available information and data, our knowledge is still limited. Most of the enzymes involved in animal fatty acid synthesis are membrane bound, which makes it extremely challenging to systematically analyze their kinetics in controlled *in vitro* experiments. For instance, the purification of such enzymes requires their solubilization, which may lead to an alteration or even a complete loss of their activity [109]. Furthermore, this step is particularly tedious, which possibly explains why most studies that we reviewed instead use recombinant proteins or cell extracts. Remarkably, recent approaches have shown successes in characterizing membrane-bound enzymes by embedding them into liposomes, mimicking their natural environment [109,131]. Even if these techniques are further developed to allow for systematic determination of kinetic parameters, it still has to be considered that an *in vitro* system never precisely reflects the situation *in vivo*. The conditions of *in vitro* assays, both physical (e.g., pH, temperature) and chemical (e.g., buffer) can influence the measured kinetic parameter values, due to suboptimal enzymatic conformation changes during the reaction. Besides, the complexity of the enzymatic process itself can limit the development of new assays. That is for instance the case of the Δ*X* desaturation process that includes a reduction step involving an extra enzyme, cytochrome *b*_5_ reductase. Furthermore, the unavailability of the purified native enzyme may lead to *in vitro* experiments based on either truncated or recombinant enzymes, or cell extracts. Still, it is unclear whether any of these substitutes can be considered as a good proxy for their native counter-part. When kinetic data are unavailable, in order to develop a mathematical model, an alternative is to use information from a distinct tissue or isoform, or an orthologous protein from a closely related organism. We showed in particular the similarities observed by Cheng et al. [66] for the values of the kinetic parameters for the ACC enzymes. Still, one cannot generalize such alikeness, and it is important to carefully consider the limits of these approximations. Although Cox and Hammes [34] provided detailed kinetic information on FAS from chicken liver, we can similarly question whether the derived parameter values reflect those in mammals. Naturally, ethical concerns restrict the possibility to perform *in vivo* experiments in humans. Therefore, alternative methods focusing on simpler systems appear as promising technologies to better mimic *in vivo* conditions. They, for instance, consist in cultivating specific cell lines (e.g., adipocytes and hepatocytes) or grow organ on chips [132,133].

It is currently feasible to build mathematical and computational models of animal fatty acid synthesis based on available kinetic information. However, literature gaps present a major obstacle for developing a more fundamental understanding of these pathways, which may considerably impair research progress, and its implications in the medical domain. To overcome these limitations, it will not be sufficient to simply perform more experiments, but it will also be necessary to find unifying standards to test, report, and store this important wealth of data in a findable and reusable manner. Additionally, we must take advantage of the growing field of targeted metabolomics, utilizing techniques such as stable isotope labeling, for measuring the kinetic parameters both *in vitro* and *in vivo*.

## Perspectives

- **Highlight importance of the field:** Fatty acid metabolism is essential for mammals and is associated with several disorders, some of which are severe societal burdens. To tackle these challenges, mathematical and computational models are increasingly important. They allow to test specific hypothesis, make predictions, rationalize experimental and medical data, and guide the design of new experiments.
- **Summary of the current thinking:** Enzymes involved in the fatty acid *de novo* synthesis have received far more attention than those in the microsomal modification. In addition, several limitations must be taken into consideration when developing a mathematical model. Most experiments are performed *in vitro*, purified native enzymes are rarely investigated, and data are so scarce that it is impossible to gather a complete set of kinetic data for a single organism.
- **Comment on future directions:** To overcome these limitations, we suggest studying enzyme kinetics using a dual approach combining both experiments and mathematical models. Furthermore, we stress the need for a systematic and standardized reporting of kinetic information, and suggest to further take advantage of the growing field of targeted metabolomics to measure kinetics both *in vitro* and *in vivo*.

## Appendix A Symbols

**Table:**

List of Symbols	
*A*	Activator
*I*	Inhibitor
*K*_m_	Michaelis–Menten constant
*K*_a_	Activation constant
*K*_i_	Inhibition constant
*k*_cat_	Turnover number
*V*_max_	Maximum velocity of the reaction
* $K_{M_{Acet}}$ *	Michaelis–Menten constant for acetyl-CoA
* $K_{M_{M\;al}}$ *	Michaelis–Menten constant for malonyl-CoA
* $K_{M_{NADPH}}$ *	Michaelis–Menten constant for NADPH
* $K_{I_{Acet}}$ *	Inhibitor constant for acetyl-CoA
* $K_{I_{M\;al}}$ *	Inhibitor constant for malonyl-CoA
*Acet*	Acetyl-CoA
*Mal*	Malonyl-CoA

## Abbreviations

ACC: acetyl-CoA carboxylase
ACP: acyl carrier protein
ADP: adenosine diphosphate
ATP: adenosine triphosphate
BAT: brown adipose tissue
ELOVL: elongation of very long-chain fatty acid
ER: endoplasmic reticulum
FA: fatty acid
FADNS: fatty acid *de novo* synthesis
FAS: fatty acid synthase
HCD: high carbohydrates diet
HUFA: highly unsaturated fatty acid
LC/MS/MS: liquid chromatography/mass spectrometry/mass spectrometry
LCFA: long-chain fatty acid
LCSFA: long-chain-saturated fatty acid
MS: mass spectrometry
MUFA: monounsaturated fatty acid
NADPH: nicotinamide adenine dinucleotide
ODE: ordinary differential equations
PPARα: peroxisome proliferator-activated receptor α
PUFA: polyunsaturated fatty acid
RF-MS: RapidFire Mass Spectrometry
SCD: stearoyl-CoA desaturase
SFA: saturated fatty acid
ULCFA: ultra-long-chain fatty acid
USFA: unsaturated fatty acid
VLCFA: very long-chain fatty acid
WAT: white adipose tissue
